# MRI and MDCT Findings of Kaposiform Hemangioendothelioma in the Oral Cavity of a Neonate: A Case Report and Literature Review

**DOI:** 10.2174/0115734056477096260525110239

**Published:** 2026-06-01

**Authors:** Yaoxi Jiang, Wei Tang, Song Peng

**Affiliations:** 1 Department of Radiology, Chongqing Health Center for Women and Children, Women and Children’s Hospital of Chongqing Medical University, Chongqing 401147, China

**Keywords:** Kaposiform hemangioendothelioma, MDCT, MRI, Prenatal diagnosis, Histopathological examination, Neonate

## Abstract

**Introduction:**

Kaposiform hemangioendothelioma (KHE) is a rare, locally aggressive vascular tumor of childhood. However, a detailed description of its imaging features, especially for neonatal oral cavity lesions, is lacking in the literature, with no prior reports detailing both prenatal magnetic resonance imaging (MRI) and postnatal multi-detector computed tomography (MDCT) findings. This report presents a case of neonatal KHE, focusing on its distinctive imaging manifestations on both modalities to provide diagnostic insights for this rare condition in an unusual location.

**Case Presentation:**

Prenatal MRI at 38 weeks of gestation and MDCT two days after birth revealed a soft tissue mass in the oral cavity protruding outward. Hemorrhage, hemosiderin deposition, and prominent flow voids within the lesion, as well as destruction or erosion of adjacent bone structures, were observed on MDCT and/or MRI. On the fifth day after birth, a biopsy of the oral mass was performed, and histopathological examination confirmed the diagnosis of KHE. Two months later, the newborn was transferred to another hospital for surgical resection of the tumor. At telephone follow-up three months after surgery, the mother reported that the child was able to swallow and feed normally.

**Conclusion:**

KHE is a rare vascular tumor that can be easily mistaken for a benign hemangioma. A definite diagnosis can be reached based on characteristic imaging findings on MDCT and MRI, particularly in newborns and children.

## 1 INTRODUCTION

Kaposiform hemangioendothelioma (KHE) is a rare and locally invasive vascular tumor, often originating from the skin and invading adjacent soft tissue and bone [1]. KHE most commonly involves the skin of the extremities, followed by the trunk, head, and neck, and rarely the abdominal cavity, retroperitoneum, and intracranial regions [2, 3]. Most cases present with skin changes, manifested as purple-blue or reddish-brown discoloration that does not fade with pressure and has ill-defined borders with surrounding tissues [4-6]. In clinical practice, KHE is prone to misdiagnosis as a common vascular tumor or malignancy [7].

Previously, cases of KHE at various anatomical sites have been reported [5, 7-8]. However, the oral cavity is an unusual location for KHE, and case reports of oral KHE are rare. To the best of our knowledge, no case report has described the prenatal MRI findings of oral KHE in a fetus. Prenatal MRI is extremely valuable for evaluating normal fetal anatomy and diagnosing fetal anomalies, and its findings can guide treatment planning, delivery management, and parental counseling [9]. The diagnosis of neonatal oral KHE is particularly challenging due to the high risk of misdiagnosis as infantile hemangioma or other vascular lesions, and the potential for life-threatening complications such as Kasabach-Merritt phenomenon and airway compromise. These challenges underscore the need for increased awareness of the imaging features of KHE in this specific population and location. In this report, we describe a case of neonatal oral KHE evaluated by prenatal MRI at 38 weeks and postnatal MDCT, and we review the characteristic imaging features, treatment, and prognosis.

## 2 CASE PRESENTATION

**Fig. (1):**
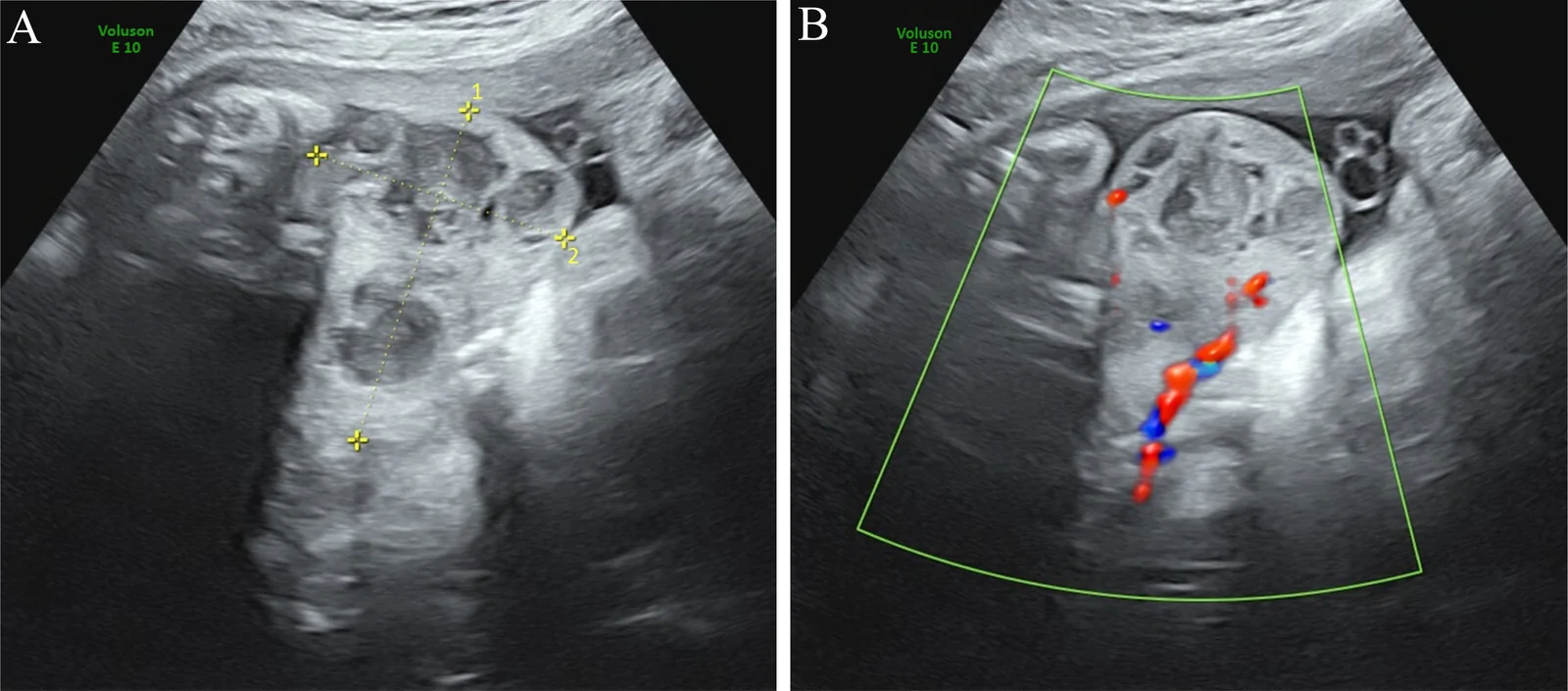
Prenatal US at 38 weeks. (**A**) Image shows a 5.0×4.1cm solid mass in the oral cavity protruding outward. (**B**) Color Doppler image demonstrates abundant internal blood flow.

**Fig. (2):**
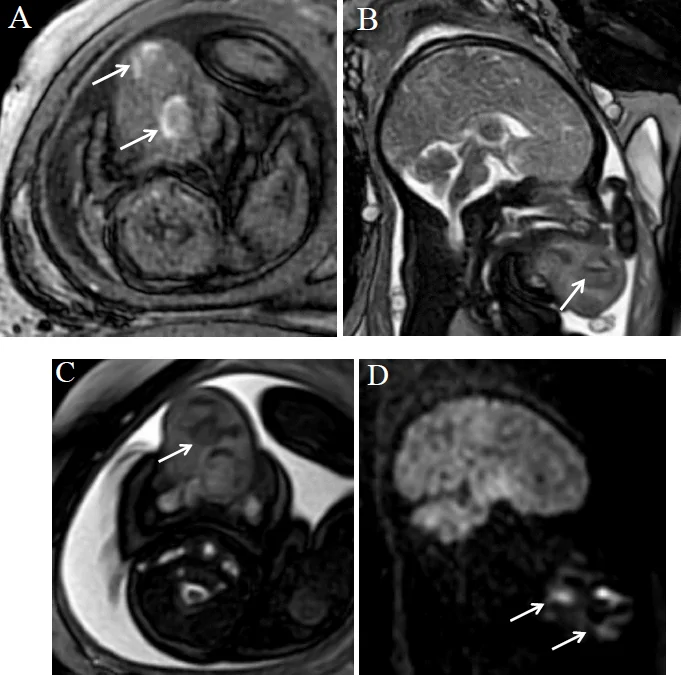
Prenatal MRI at 38 weeks. (**A**) Axial T1-weighted image shows a mass isointense to muscle with patchy hyperintense areas (arrow). (**B**, **C**) Sagittal and axial T2-weighted images show a mass slightly hyperintense to muscle with patchy hypointense areas (long arrow). (**D**) Sagittal diffusion-weighted image shows slightly heterogeneous hyperintense signal (arrow).

A 38-year-old multiparous woman was referred at 38 weeks of gestation after ultrasonographic diagnosis of a fetal intraoral mass. Prenatal ultrasound showed a heterogeneous solid mass in the fetal oral cavity protruding outward, measuring approximately 5.0 × 4.1 cm (Fig. **1A**), with abundant internal vascularity (Fig. **1B**). On the same day, fetal MRI revealed a soft tissue mass measuring approximately 5.2 × 4.2 cm. The mass was isointense to muscle on T1-weighted images with patchy hyperintense areas (Fig. **2A**), slightly hyperintense on T2-weighted images with patchy hypointense areas (Fig. **2B** and **C**), and slightly heterogeneous hyperintense on diffusion-weighted images (Fig. **2D**). The posterior margin of the lesion was indistinct from the tongue.

The mother denied any history of viral infection, toxin exposure, or use of drugs or radiation during pregnancy. Early in the pregnancy, she was diagnosed with hypothyroidism and treated with levothyroxine, which normalized her thyroid function. Two days before cesarean section, she was hospitalized for elevated blood pressure (147/98 mmHg) and received magnesium sulfate for blood pressure control. She had previously undergone a cesarean section in 2011 and delivered a healthy female infant.

On the second day after prenatal imaging, a boy was delivered by cesarean section at 38 weeks of gestation (birth weight 3000 g, length 51 cm) with Apgar scores of 9 at 1 minute and 9 at 5 minutes. The umbilical cord was wrapped once around the neck. Physical examination revealed an ill-defined, reddish-blue soft tissue mass measuring approximately 4 × 4 × 3.5 cm at the anterior tongue, extending outside the oral cavity and preventing lip closure. Mild tachypnea without progressive respiratory distress was noted, and the newborn was admitted to the neonatology department for nasal continuous positive airway pressure (nCPAP) with FiO_2_ 30% and PEEP 3 cm H_2_O.

**Fig. (3):**
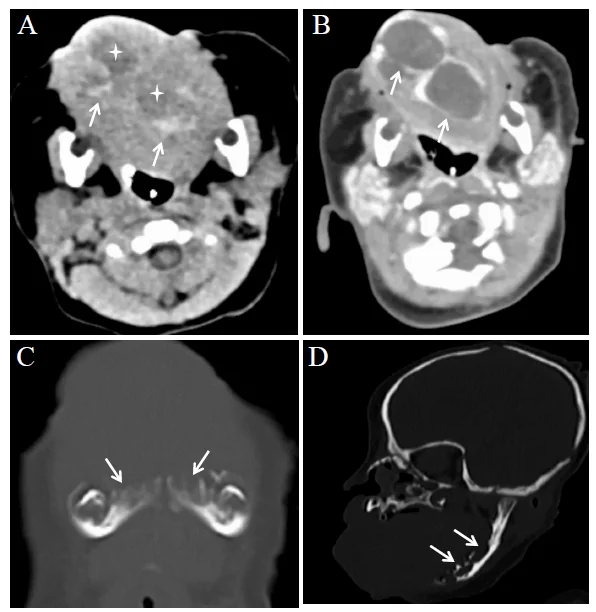
Postnatal MDCT at 2 days old. (**A**) Axial non-contrast CT shows an inhomogeneous mass with linear hyperdense areas (arrow) and multiple cystic hypodense areas (cross star). (**B**) Axial contrast-enhanced CT shows heterogeneous enhancement with rounded enhancing areas (arrow). (**C**, **D**) Axial and sagittal bone window images demonstrate bone resorption of the mandible adjacent to the mass (arrow).

Two days after birth, non-contrast and contrast-enhanced MDCT of the head and neck was performed. Non-contrast images showed an inhomogeneous soft tissue mass in the oral cavity with linear hyperdense areas and multiple cystic hypodense areas (Fig. **3A**). The mass extended from the tongue posteriorly to outside the oral cavity anteriorly, measuring approximately 4.3 × 5.8 cm. Contrast-enhanced images demonstrated heterogeneous enhancement with rounded enhancing areas (Fig. **3B**). In addition, bone resorption of the adjacent mandible was noted (Fig. **3C** and **D**). Quantitative MDCT analysis revealed mean CT values of 56 HU for the solid portion on non-contrast images and 126 HU on contrast-enhanced images, with a net enhancement of 70 HU and a lesion-to-muscle enhancement ratio of 1.35, confirming the hypervascular nature of the lesion.

On admission, laboratory tests showed: white blood cell count 13.5 × 10^9^/L, hemoglobin 178 g/L, hematocrit 51.5%, platelet count 49 × 10^9^/L, activated partial thromboplastin time 43.8 s, international normalized ratio (INR) 1.17, prothrombin time 13 s, thrombin time 20 s, fibrinogen 1.6 g/L, fibrinogen degradation products 21.9 mg/L, and D-dimer 7.74 mg/L.

**Fig. (4):**
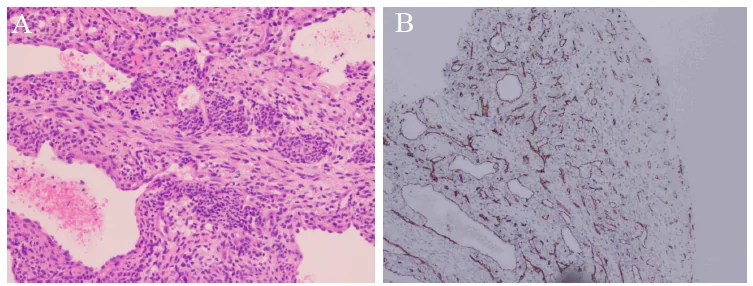
Histopathology of the oral mass. (**A**) Hematoxylin and eosin staining (×200) shows a glomeruloid pattern with central epithelioid cells surrounded by spindle cells, slit-like vessels, hemosiderin deposition, and extravasated red blood cells. (**B**) Immunohistochemical staining (×200) shows positive CD34 expression in spindle-shaped endothelial cells.

On the fifth day after birth, a biopsy of the oral mass was performed. Under sterile conditions, a biopsy needle was inserted approximately 2.5 cm into the mass, and a 2 × 0.2 × 0.2 cm tissue specimen was obtained. Histopathological examination showed infiltrating nodules composed of spindle-shaped endothelial cells, and immunohistochemical staining demonstrated positive expression of CD34 in these cells (Fig. **4A** and **B**). Based on these findings, the diagnosis of Kaposiform hemangioendothelioma (KHE) was confirmed.

Due to neonatal thrombocytopenia, oral sirolimus was administered at a dose of 0.083 mg every 12 hours. Blood drug concentrations were monitored regularly. During hospitalization, the infant's condition improved, with the platelet count increasing to 95 × 10^9^/L and the oral mass decreasing in size. Two months later, the infant was transferred to another hospital for surgical resection of the residual tumor. Three months after surgery, during a telephone follow-up, the mother reported that the child was able to swallow and feed normally.

## 3 DISCUSSION

Kaposiform hemangioendothelioma (KHE) was first described by Zukerberg *et al*. in 1993 [10]. It is a rare vascular tumor that primarily affects infants and young children, presenting as an infiltrative, erythematous, or purpuric soft tissue mass involving the skin, deep soft tissues, or viscera [11, 12]. Oral cavity involvement is exceptionally rare. A 2021 review identified only four reported cases in the oral cavity and oropharynx [12], with additional isolated cases reported subsequently at various subsites [13-17]. These cases demonstrate that oral KHE, while predominantly affecting infants and young children, can occasionally occur in adults, with no apparent gender predilection. In the present study, we describe a rare case of neonatal KHE arising from the tongue and evaluated by both prenatal MRI and postnatal MDCT-a combination not previously reported.

Our patient underwent three imaging modalities: prenatal ultrasound, prenatal MRI, and postnatal MDCT. Ultrasound provided an initial assessment of lesion size, margins, and vascularity, but was limited in evaluating the relationship with adjacent structures. In contrast, MRI and MDCT offered more detailed information, including involvement of surrounding tissues, internal hemorrhage, and feeding or draining vessels [18].

A combined analysis of MDCT and MRI findings in this case provided complementary information that enhanced diagnostic accuracy. MDCT clearly demonstrated bone changes, showing resorption of the mandible adjacent to the mass (Fig. **3C** and **D**, arrow)-a key feature of the locally infiltrative nature of KHE. Regarding hemorrhage, both modalities contributed valuable information: MDCT showed linear hyperdense areas on non-contrast images (Fig. **3A**, arrow), while MRI confirmed hemorrhage with corresponding hyperintense signal on T1WI (Fig. **2A**, arrow), indicating methemoglobin from subacute bleeding. Notably, MRI uniquely identified a hypointense signal on T2WI surrounding the lesion and cystic components (Fig. **2B**, arrow). This finding reflects hemosiderin deposition and a dense hyaline stromal response [5, 19]-histopathological hallmarks of KHE not visible on CT. Multiple cystic components were present within the lesion, with the two largest measuring approximately 2 cm in diameter. These cystic structures were well depicted on both modalities: on CT, they appeared as hypodense areas with enhancing walls (Fig. **3A** and **B**); on MRI, they demonstrated T1-hyperintense (hemorrhagic) walls and T2-hypointense (hemosiderin-laden) rims (Fig. **2A** and **B**). Together, these multimodality features-bone erosion (CT), hemorrhage (CT and MRI), and T2 hypointensity reflecting hemosiderin (MRI)-form a constellation of findings strongly supporting the diagnosis of KHE and aiding in its distinction from other vascular lesions. Quantitative MDCT analysis further confirmed the hypervascular nature of the lesion, with a net enhancement of 70 HU and a lesion-to-muscle enhancement ratio of 1.35.

These imaging findings correlate directly with the histopathological features of KHE. The hypointense signal on T2WI corresponds to hemosiderin deposition (Fig. **4A**)-a characteristic finding that distinguishes KHE from other vascular tumors. The hemorrhagic components seen on MRI (T1 hyperintensity) and MDCT (hyperdense areas) correlate with extravasated red blood cells (Fig. **4A**), reflecting the bleeding tendency of KHE. The enhancing areas on MDCT correspond to the glomeruloid pattern and slit-like vascular channels (Fig. **4A** and **B**), representing the abnormal vasculature. The ill-defined margins and bone erosion seen on imaging are consistent with the infiltrative growth pattern of spindle-shaped endothelial cells (Fig. **4A**). This imaging-pathology correlation validates the imaging findings and reinforces the diagnosis of KHE.

Due to abnormal capillary endothelial cell proliferation, up to 70% of patients develop Kasabach-Merritt phenomenon (KMP) [20]. KMP is diagnosed when the platelet count falls below 100 × 10^9^/L, with or without hypofibrinogenemia and elevated D-dimer [21]. According to these criteria, our patient was diagnosed with KHE associated with KMP. Hauer *et al*. [22] reported that the occurrence of KMP is related to patient age and lesion size but not to tumor location. Younger age and larger lesion diameter are associated with a higher incidence of KMP. The lesion size and age of our patient are consistent with these observations, suggesting a high likelihood of KMP.

This association with KMP, combined with the atypical oral location, underscores the diagnostic challenge of this case. Accurate recognition of KHE is critical, as its management differs from that of other neonatal oral vascular lesions. Unlike infantile hemangioma, which typically presents as a well-defined mass with homogeneous T2 hyperintensity and lacks intralesional hemorrhage or bone involvement, our case demonstrated ill-defined margins, T2 hypointensity reflecting hemosiderin deposition, hemorrhagic components, and adjacent bone erosion-features characteristic of KHE. In contrast to lymphatic malformations, which appear as cystic lesions with T2 hyperintensity and absent enhancement, our case showed a solid enhancing mass with internal hemorrhage. Congenital hemangioma and venous malformations may also present at birth as vascular masses, but they lack the infiltrative growth pattern, progressive thrombocytopenia, and osseous erosion observed in this case. Familiarity with these distinguishing imaging features, combined with clinical awareness of KMP, aids in accurate diagnosis, timely intervention with sirolimus, and informed parental counseling regarding potential complications.

The management of KHE with KMP is a critical clinical challenge. In this case, the tumor originated from the tongue with ill-defined posterior margins, and severe thrombocytopenia (platelet count 49 × 10^9^/L) precluded immediate surgical resection. Sirolimus, an mTOR pathway inhibitor, is preferred for KHE with KMP due to its dual mechanism: it controls tumor growth by inhibiting angiogenesis and lymphangiogenesis while improving thrombocytopenia by reducing platelet trapping within the tumor [23]. Recent studies recommend a target trough concentration of 5–10 ng/mL with regular monitoring in neonates [24]. The newborn received sirolimus 0.083 mg every 12 hours during hospitalization with regular blood drug monitoring. The treatment resulted in platelet count improvement (to 95 × 10^9^/L) and tumor shrinkage, with no significant adverse events. In this case, sirolimus served as an effective bridge to surgery, enabling hematologic stabilization and tumor reduction before definitive resection.

## CONCLUSION

KHE is a rare vascular tumor that can be easily mistaken for a benign hemangioma, particularly in neonates. Early and accurate imaging diagnosis is crucial, as it enables timely intervention, reduces the risk of life-threatening complications such as Kasabach-Merritt phenomenon, and guides appropriate management strategies. Recognition of characteristic imaging features-including T2 hypointensity reflecting hemosiderin deposition, hemorrhagic components, and adjacent bone erosion-can help clinicians distinguish KHE from other vascular lesions and improve clinical outcomes.

## Data Availability

All data generated or analyzed during this study are included in this article. Further inquiries can be directed to the corresponding author [W.T] upon reasonable request.

## LIST OF ABBREVIATIONS

CD34: Cluster of differentiation 34
KHE: Kaposiform hemangioendothelioma
KMP: Kasabach-Merritt phenomenon
MDCT: Multi-detector computed tomography
MRI: Magnetic resonance imaging
T1WI: T1-weighted image
T2WI: T2-weighted image
US: Ultrasound

## STANDARDS OF REPORTING

CARE guidelines were followed.
